# Effect of the electrochemical characteristics of titanium on the adsorption kinetics of albumin

**DOI:** 10.1039/c9ra05988a

**Published:** 2019-10-24

**Authors:** Evangelos Liamas, Owen R. T. Thomas, Anna Igual Muñoz, Zhenyu J. Zhang

**Affiliations:** School of Chemical Engineering, University of Birmingham Edgbaston Birmingham B15 2TT UK z.j.zhang@bham.ac.uk; Department of Chemical and Nuclear Engineering, Universidad Politécnica de Valencia Valencia E-46071 Spain anna.igualmunoz@epfl.ch; School of Engineering, Materials Science and Engineering, EPFL MXC 341 (Bâtiment MXC), Station 12 CH-1015 Lausanne Switzerland

## Abstract

An electrochemical quartz crystal microbalance (EQCM) was used to examine the electrochemical behaviour of pure titanium in phosphate buffered saline (PBS) and PBS-containing bovine serum albumin (BSA) solutions, and the associated adsorption characteristics of BSA under cathodic and anodic applied potentials. It was found that the electrochemical behaviours of bulk titanium substrate and titanium-coated QCM sensors are slightly different in PBS buffer solution, which is attributed to the difference in their surface roughness. The oxide film formed on the surface of the QCM sensor during potentiostatic tests was found to affect its electrochemical behaviour, while cathodic cleaning is not sufficient to have it removed. Lastly, the excessive amount of electrons on the titanium surface upon application of a cathodic potential could result in the desorption of BSA due to electrostatic repulsion and protein dehydration. In contrast, application of anodic potential charges the titanium surface positively and can facilitate protein adsorption when the surface is not saturated with protein.

## Abstract 2

An EQCM was used to examine the electrochemical behaviour of pure titanium in PBS and PBS-containing BSA solutions, and the associated adsorption characteristics of BSA under cathodic and anodic applied potentials.

## Graphical abstract

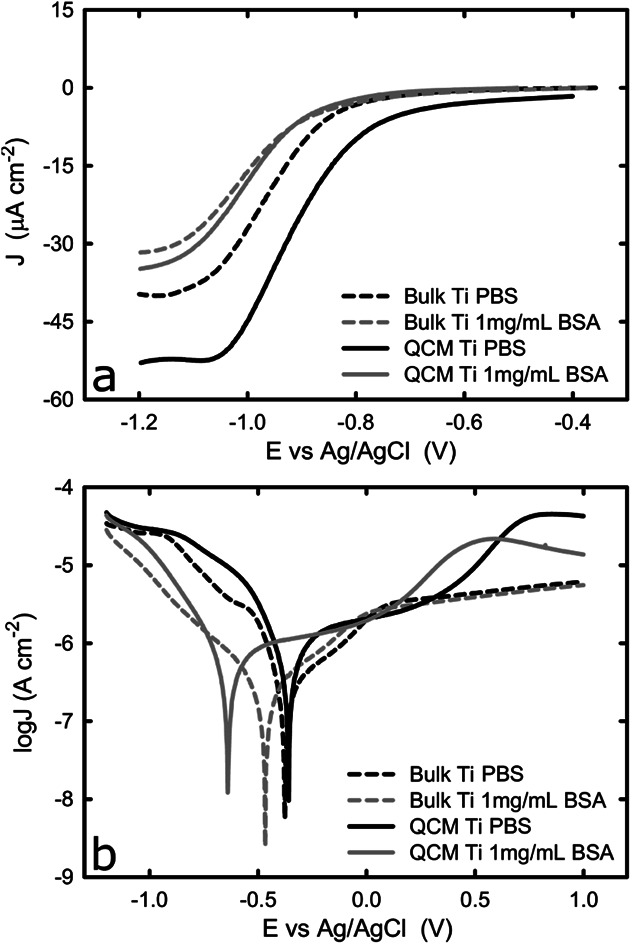

## 1 Introduction

Titanium is a widely used biomaterial in dental and orthopaedic applications due to its unique combination of physical properties and corrosion resistance.^1–3^ Similar to that of most metallic materials used for bio-applications, corrosion resistance of titanium and its alloys is highly dependent on the passive film developed on the surface.^4^ When a passive metal, such as titanium, is exposed to air or liquid, an oxidation reaction takes place, resulting in the formation of a thin metal oxide with thickness of a few nanometres. This oxide film not only protects the underlying material from corrosion, but also provides an excellent interface to interact with the surrounding tissue due to its biocompatibility and osseointegration.^5–7^ Therefore, an in-depth understanding of the factors that determine the stability of the oxide film, particularly when being exposed to a physiological environment, is vital for producing novel biomaterials with enhanced properties.

Upon contact with biological fluids, the surface of an implant is covered with a dynamic film of proteins that dictates the initial cellular and, subsequently, the host response. Consequently, proteins play a key role in the integration of implants with the surrounding tissue as it is the first step in the cell adhesion process. Since cells cannot adhere directly with the surface of an implant but instead through an intermediate interaction of adsorbed proteins, their interaction with passive films is an important factor that dictates the biocompatibility of a biomaterial.^8,9^ Albumin is the most abundant protein found in body fluids and has been studied extensively.^10^ It can be found primarily in serum or synovial fluid with a concentration of 37.6–54.9 mg mL^−1^ and 6–10 mg mL^−1^ respectively.^11^ It has a molar mass of approximately 65 kDa and is consisted of 585 residues that are organised in a highly helical structure with three sub-domains.^12^ Due to its helical structure, albumin has high flexibility allowing a rapid expansion and contraction. Upon adsorption on solid substrates, the dimensions of albumin are ranging from 9.2 × 9.2 × 3.0 nm (triangular shape) on surfaces with low adhesion, to 25.0 × 2.1 × 2.1 nm (elongated shape) on surfaces with larger adhesion.^13^ In comparison, the hydrodynamic radius, *R*_H_, of BSA in solution is ranging from 3.3 to 4.3 nm.^14^ The isoelectric point of albumin is 4.9, which means that it has an overall negative charge at pH = 7.^15^

Under mechanical impact, such as that occurring at a sliding interface of a hip-joint implant, the passive film could break down and release metal ions into the surrounding environment, *e.g.* the tissue in contact. This happens by active dissolution and could be affected by the presence of proteins. Consequences of releasing metal ions to the surrounding tissues range from discolouration to more severe biological reactions, such as cytotoxicity and tissue necrosis.^16,17^ Furthermore, the tissue in the areas of corrosion could recede, forming small pockets that facilitate the formation of a micro-environment suitable for bacterial and viral infections.^12^ As a result, the film of adsorbed protein surrounding the implant could be infected, which in turn accelerates the corrosion process as a result of the metabolic product of the presence of bacteria, pH changes, and increases of local temperature.^12^ Therefore, understanding the effect of proteins on the electrochemical properties of passive film, as well as their interaction, is of significant importance.

There have been several studies concerning the effect of proteins on the electrochemical behaviour of metal surfaces. It was found that on CoCrMo surfaces, in phosphate buffer saline (PBS) or sodium chloride (NaCl) solutions, BSA acts as a cathodic inhibitor accelerating the anodic reaction and shifting the corrosion potential towards more cathodic values.^18^ Consistent results were reported when titanium alloys were exposed to PBS solution in the presence of BSA or collagen.^19^ It was suggested that protein molecules adsorb onto surface sites where oxygen reacts with electrons available on the surface, and consequently block the cathodic reaction. Furthermore, on Ti-6Al-4V surfaces, it was found that the presence of BSA in simulated inorganic plasma solutions is reducing the passive current density, and thus lowering the corrosion rate, while on platinum surfaces the adsorption of proteins such as insulin, myoglobin, casein, fibrinogen, and BSA could inhibit the growth of surface oxide layer at anodic conditions.^20,21^ On stainless steel surfaces, the reported results are less consistent. While BSA diluted in PBS acts as cathodic inhibitor, BSA diluted in NaCl has the opposite effect since it acts as an anodic inhibitor and accelerates the metal dissolution.^18^ Another study showed that the adsorption of BSA on stainless steel decreases the corrosion activation energy at anodic conditions, which could be due to the negatively charged carboxylate groups of the proteins that act as anchoring sites during the adsorption onto stainless steel – a process that is accompanied by transfer of charge.^22^ This is in agreement with another study concerning the adsorption of BSA and fibrinogen on titanium surfaces where the surface charge density was found directly proportional to the amount of adsorbed protein.^12^ The same study suggests that the adsorption process is endothermic and entropically controlled, implying that the interaction between protein and titanium surface is chemisorption, which leads to denaturation of the protein. Similar results, denaturation of protein, was also shown on globular proteins upon adsorption onto the electrode surface of platinum.^23^ In conclusion, although a number of mechanisms are associated with the interaction between proteins and solid substrates, it has been shown that BSA generally acts as a cathodic inhibitor but can also increase the dissolution rate of materials.

To examine the adsorption process of protein under controlled electrochemical conditions, Electrochemical Quartz Crystal Microbalance (EQCM) has been used widely as it not only quantifies the adsorption kinetics of proteins on selected substrates *in situ* with sub-monolayer sensitivity, but also reveals the fine details related to the protein–surface interactions with modification to the passive film.^24^ The device is comprised of a quartz crystal that is sandwiched between electrodes and driven at its resonance frequency. Any variation in the mass sensed by the surface, due to protein adsorption/desorption, results in a shift in the resonance frequency that can be converted to mass using the Sauerbrey equation.^25^ EQCM has been employed in the past to study the oxidation and the dissolution of metals. For instance, a study on CoCrMo surface has showed a built up of oxide film under low cathodic conditions, followed by a monotonic loss of mass during the passive domain and a large decrease of mass at the transpassive domain due to transpassive dissolution.^26^ Another EQCM study of pure titanium in sulphuric acid showed that while moving from a cathodic region up to the passive region a loss in mass was observed as a result of passive dissolution of titanium, while an increase in mass was observed over the passive region for potentials up to 2 V.^27^

The aim of the present study is to investigate the corrosion behaviour of pure titanium and to understand the effect of BSA on its electrochemical behaviour. For this reason, electrochemical quartz crystal microbalance was used allowing for the quantification of the passive film growth onto the surface of titanium, as well as the effect that BSA has on its passive film dissolution. The adsorption of bovine serum albumin onto pure titanium surfaces has also being investigated under different applied potentials. The results of this study are expected to provide a significant insight on the electrochemical processes occurring on sliding titanium interfaces and lead to development of improved biomaterial coatings.

## 2 Materials and methods

### 2.1 Materials

Pure titanium substrates (purity 99.99+%) were purchased from Goodfellow Cambridge Ltd (Huntingdon, UK). Titanium coated quartz crystals (purity 99.995%, AT-cut, 5 MHz, 1 inch diameter) were purchased from Testbourne Ltd (Basingstoke, UK). Silicon nitride atomic force microscope (AFM) cantilevers were purchased from BudgetSensors (Sofia, Bulgaria). Fatty acid free (>99%) bovine serum albumin (BSA) was purchased from Sigma-Aldrich Company Ltd (Dorset, UK). Phosphate buffer saline (PBS) and sodium dodecyl sulphate (SDS) were purchased from Fisher Scientific (Loughborough, UK).

### 2.2 Sample preparation

Titanium sample (25 mm by 25 mm) was wet-ground with 500 to 4000 grit SiC paper, and was further polished with OP-Chem polishing cloths using 1 μm diamond particles to achieve a mirror like finishing. Following the polishing process, and prior to each test, the samples were cleaned in ultrasonic baths of sodium dodecyl sulphate (SDS) solution and distilled water for 10 min respectively to remove any residual particles or protein from the surface. The samples were then dried by a stream of compressed air. The same procedure was used to clean the QCM crystals. BSA solutions were prepared using the PBS buffer to obtain protein concentrations of 1 mg mL^−1^ and 10 μg mL^−1^.

### 2.3 Electrochemical measurements on titanium

All electrochemical measurements were controlled using a PSTAT 302N potentiostat (Metrohm Autolab B.V., Utrecht, Netherlands). The setup is a conventional three-electrode electrochemical cell with a platinum wire as a counter electrode and an Ag/AgCl (3 M KCl) reference electrode. All potentials were referred to the reference electrode (0.205 V *versus* standard hydrogen electrode, SHE). The adsorbed mass was measured using an electrochemical quartz crystal microbalance (EQCM) by Inficon Holding AG (Bad Ragaz, Switzerland). The topographic images were acquired in liquid tapping mode using a Nanowizard II atomic force microscope (JPK Instruments AG, Berlin, Germany).

The same setup was used to compare between bulk titanium substrate and titanium-coated sensor. The bulk titanium specimen was mounted on a flat cell with its lower part insulated and connected to the potentiostat (working electrode), while its upper surface (0.785 cm^2^) was exposed to 40 mL of the electrolyte solution. Similarly, a titanium-coated QCM crystal was loaded in the EQCM holder and immersed into 40 mL of the electrolyte solution, leaving its upper surface (1.332 cm^2^) exposed. In both cases, the reference electrode was no further than 1 cm away from the surface. The experiments were carried out in two different electrolytes: (i) 1× PBS solution and (ii) 1 mg mL^−1^ BSA in 1xPBS. Two different electrochemical tests were conducted on the titanium surface: potentiodynamic curves and potentiostatic tests.

To generate the potentiodynamic curves, a cathodic cleaning was performed by applying −1200 mV_Ag/AgCl_ for 300 seconds after the immersion of titanium in the electrolyte. This procedure removes any oxide species from the surface and ensures good reproducibility for the following experiments. The open circuit potential (OCP) was then measured for 30 min. Upon equilibrium, the potential was moved cathodically from OCP to −1200 mV_Ag/AgCl_ with a scan rate of 2 mV s^−1^ and then anodically to +1000 mV_Ag/AgCl_ with the same scan rate, while registering the response in current.

For potentiostatic tests, the procedure started with a cathodic cleaning for 300 seconds at −1200 mV_Ag/AgCl_ and measurement of the OCP for 30 min. Subsequently, the potential moved cathodically to −1200 mV_Ag/AgCl_, or anodically to +1000 mV_Ag/AgCl_, with a scan rate of 100 mV s^−1^. Potentiostatic tests were carried out at the reported potentials for 30 min, registering the response in current whilst measuring the change in mass on the crystal.

## 3 Results and discussion

### 3.1 Corrosion mechanisms

**Fig. 1:**
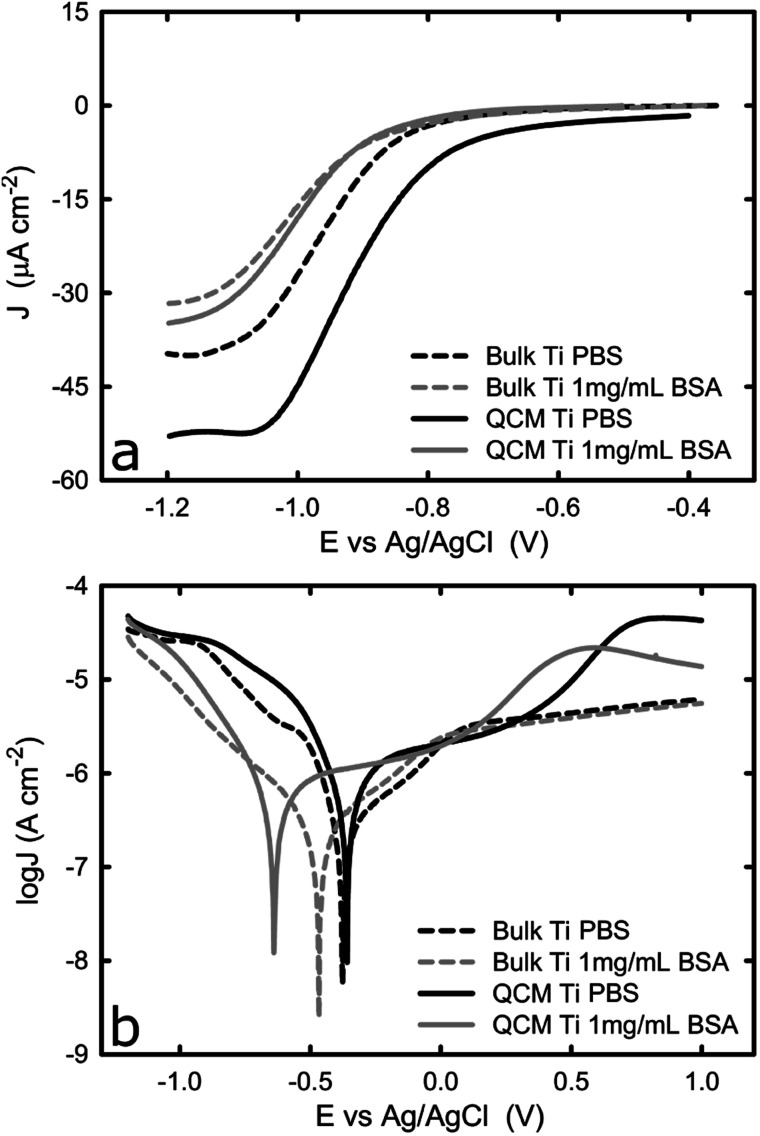
(a) *i*–*E* curves of bulk and new titanium coated QCM crystals in 1× PBS with and without BSA, and (b) potentiodynamic curves of bulk and new titanium coated QCM crystals in 1× PBS with or without BSA. The graphs represent the average values of *n* = 3 measurements.

To ensure the electrochemical properties of the titanium-coated QCM sensor are comparable to that of bulk titanium, potentiodynamic tests in PBS solution were performed on both samples, with or without BSA. This would verify whether the titanium-coated sensor is a representative system for bulk titanium products that are commonly used in biomedical devices. Fig. 1a shows the representative potentiodynamic curves acquired from one bulk titanium and a new titanium-coated QCM crystal in PBS with or without BSA. During the cathodic scan, the cathodic current density was found to remain unchanged until approximately −0.8 V_Ag/AgCl_, and then increase (in absolute value) linearly until it reached a plateau at approximately −1.1 V_Ag/AgCl_. The plateau is a result of the dominant mechanism responsible for current corrosion on titanium, in the cathodic region studied here, which is the reduction of dissolved oxygen and water reduction (reactions (1) and (2)).^28,29^

1 O_2_ + 2H_2_O + 4e^−^ → 4OH^−^ 0.196 *E*^0^/*V*_Ag/AgCl_

2 2H_2_O + 2e^−^ → H_2_ + 2OH^−^ −1.033 *E*^0^/*V*_Ag/AgCl_

Because the reduction reaction takes place on the titanium surface, the oxygen molecules have to be transported from the bulk solution to the metal/liquid interface where they could interact with electrons. As the surface potential of the titanium becomes more negative, the rate of the reduction reaction increases linearly because electrons are transported faster from the bulk metal to the metal/liquid interface. Eventually, the rate of the reduction reaction reaches a maximum because it is limited by the rate of oxygen molecules being transported onto the solid/liquid interface. During this plateau phase, as was shown in Fig. 1a, the mass transport of the oxidant (oxygen) to the titanium surface is expected to limit the current, which is also confirmed by another study.^10^

**Fig. 2:**
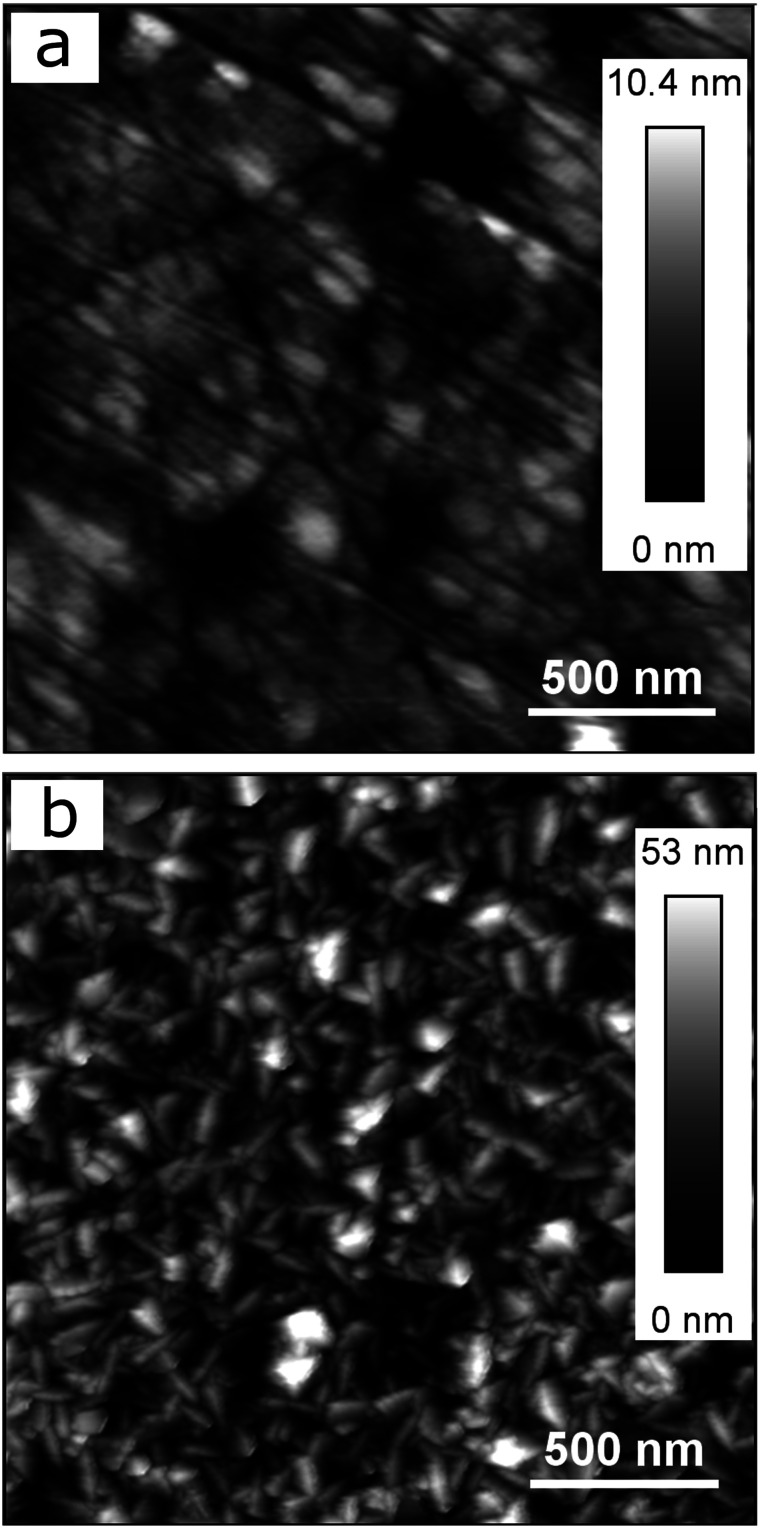
AFM images of (a) bulk titanium (*R*_a_ = 1.27 nm), and (b) titanium-coated QCM sensor (*R*_a_ = 8.78 nm).

Fig. 1a also shows that the titanium-coated QCM sensor possesses a slightly increased (in absolute value) current density in comparison to bulk titanium. It is probably caused by the differences in surface roughness between the two samples (attributed to a larger true surface area) as has been shown in previous studies.^30,31^ This is shown in Fig. 2, where the QCM surface has a larger average surface roughness (*R*_a_) than bulk titanium (*R*_a_ = 8.78 nm for titanium-coated QCM sensor *versus R*_a_ = 1.27 nm for bulk titanium). Consequently, because the true surface area is larger than the apparent area and increases with an increase in the average surface roughness, the cathodic reactions on the QCM sensor are taking place over a larger area which resulting in increased current density.

The difference in current density is, however, less significant in the BSA-containing solution, which is probably due to the adsorbed proteins reducing the amount of active sites for oxygen reduction. The cathodic current density was found to decrease (in absolute value) in both specimens in the presence of BSA, while the plateau was shifted towards more cathodic values. The general trend of the curve (Fig. 1a) acquired in the BSA solution is very similar to that collected in PBS. As the potential moves towards more cathodic values, the current density initially shows a slight increase and then an exponential increase before it reaches a plateau at around −1 V_Ag/AgCl_. The reduction in current density due to the presence of BSA is consistent with previous work where the effect of BSA on titanium alloys and CoCrMo surfaces was studied. It was suggested that a surface adsorbed protein film, such as albumin or collagen, could hinder the transport of oxygen on to the titanium surface and consequently inhibit the cathodic reaction for oxygen^13,18,19^ This is further supported by the shift of corrosion potential towards more cathodic values in the presence of BSA (Fig. 1b).

The reverse potentiodynamic scan is shown in Fig. 1b. On the cathodic domain, from −1.2 V_Ag/AgCl_ until the corrosion potential, the current density is generated by the reduction of water and oxygen. At the corrosion potential, the rate of anodic and cathodic reactions become equal and the transition from cathodic to anodic potential takes place at approximately −0.4 V_Ag/AgCl_ in PBS for both bulk titanium and QCM crystal. The presence of BSA causes the corrosion potential to shift towards more cathodic values for both types of samples, despite that the shift was greater for the Ti QCM crystal than for the bulk titanium. The shift in corrosion potential is because the adsorbed BSA inhibits the cathodic reaction, as described earlier. As the potential increases above the corrosion potential, the titanium anodically oxidizes and starts the formation of the passive oxide layer, as reaction 6 below show.^32^

3 Ti → Ti^4+^ + 4e^−^

4 Ti^4+^ + 2H_2_O → TiO_2_ + 4H^+^

5 Ti + 2H_2_O → TiO_2_ + 4H^+^ + 4e^−^

The current density continues to increase as the metal surface reacts with water to form a thin oxide film that covers the entire surface over time. The formation of such passive film slows down the rate of metal dissolution.^33^ After this point, the migration of metal ions, and thus the current, becomes independent of the potential and remains reasonably constant once the thickness of the passive film reaches its maximum value. However, while the passive domain for the bulk titanium substrate is extended up to +1.0 V_Ag/AgCl_, a different behaviour is observed for the titanium-coated QCM sensors where, following a passive domain, an increase of the current density is observed at approximately 0.4 V_Ag/AgCl_. This increase in current is probably due to a phase transformation as explained in paragraph 3.2.

As suggested by Fig. 1b, the current density acquired from bulk titanium and titanium QCM crystal in the passive region is very close, with or without the presence of BSA. This confirms that the formed oxide layer during the passivation possesses similar thickness for titanium of different physical forms in both types of specimen and, thus, titanium-coated QCM has similar electrochemical properties to bulk titanium.

### 3.2 Effect of the number of potentiodynamic scans on the electrochemical behaviour of QCM-titanium

**Fig. 3:**
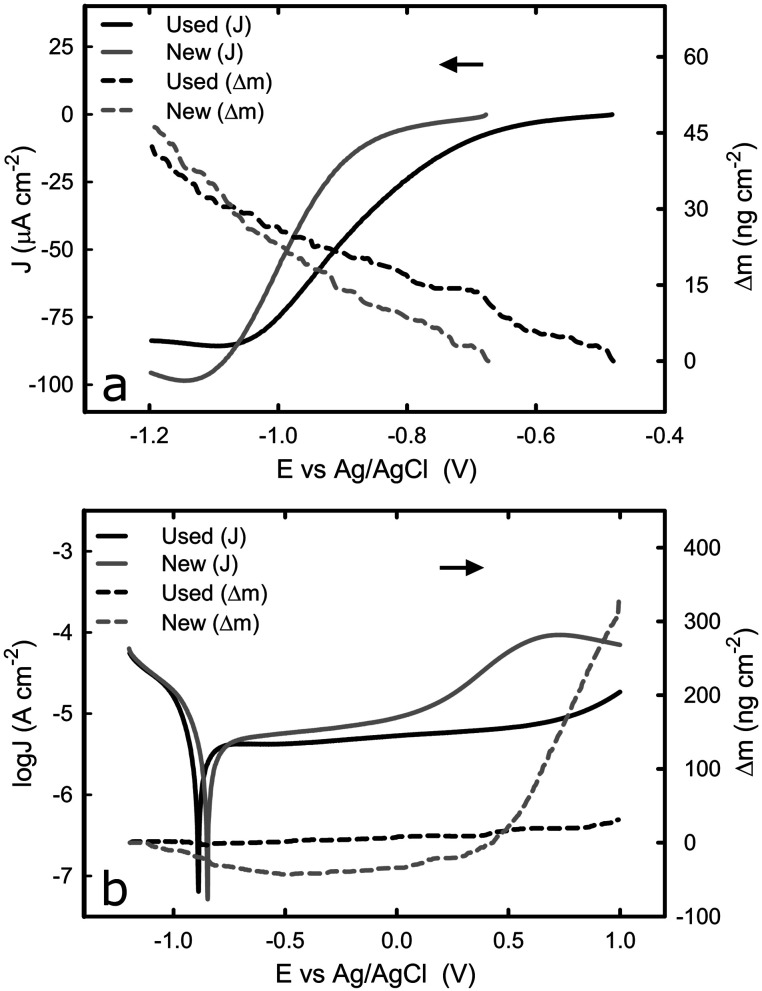
Comparison between a new titanium coated QCM crystal and the same crystal after the initial run in 1× PBS. (a) *i*–*E* curves and change of mass during transition from OCP to −1.2 V, (b) potentiodynamic curves and change of mass during transition from −1.2 V to +1.0 V. The arrows indicate the direction of the scan.

Unlike the consistent potentiodynamic results acquired from the bulk titanium samples over successive tests, there appeared some variation in the electrochemical behaviour of the titanium-coated QCM sensor over multiple potentiodynamic scans. To establish the cause of such variation, and identify the effect of the electrochemical process on the QCM sensor, EQCM was employed to monitor the mass change at the sensor surface whilst replicating the same potentiodynamic tests. Fig. 3a shows the cathodic potentiodynamic curves of a “new” (tested just after the cleaning procedure) titanium-coated QCM sensor and the same QCM sensor “used” on the second repeating potentiodynamic scan from OCP to −1.2 V_Ag/AgCl_. The current density response of both electrodes in the region from OCP to −0.85 V_Ag/AgCl_ is relatively low, and it decreases rapidly until −1.1 V_Ag/AgCl_, before reaching a plateau. Although the characteristics of potentiodynamic curves acquired from the used sensor are similar to that of new sensor, the current density at the plateau is slightly higher (in absolute value) in the new than that in the used sensor, which could be attributed to a thicker passive film on the used sensor that was acquired by passivation during the first scan. It is known that oxide film reduces the kinetics of the oxygen reduction reaction.^34^ The mass change on titanium surface (Δ*m*) over the scan was measured by the EQCM by converting the change in frequency to mass variation based on Sauerbrey equation. For both specimens, the Δ*m* was equal and approximately 40 ng cm^−2^, as shown Fig. 3a. The total mass gain during this transition is probably due to adsorption of species, such as ions, on the titanium surface. Such behaviour has been reported in the past where ions adsorbed onto gold surface.^35^ Increased concentration of ions could change the viscosity of the liquid in contact with the sensor surface, which consequently alter the frequency signal detected, as reported previously.^36,37^

The potentiodynamic curves for the reverse scan, moving anodically from −1.2 V_Ag/AgCl_ to +1.0 V_Ag/AgCl_, are shown in Fig. 3b. As expected, all three distinguished domains, including the cathodic domain from −1.2 V_Ag/AgCl_ until the corrosion potential, the transition from cathodic to anodic at the corrosion potential, and the passive domain are visible in both cases (new and used crystal). Although the corrosion potential was found to be around −0.4 V_Ag/AgCl_ and −0.8 V_Ag/AgCl_ for the same system, as shown in Fig. 1b and 3b respectively, such variation was caused by the different scan rate of the potential employed (2 mV s^−1^ in the first case *versus* 10 mV s^−1^ in the latter), which has been reported in the past.^38^ The current density of the new sensor at the passive domain is slightly higher than that of the used one, which could be attributed to a thicker passive film on the used sensor that was acquired by passivation during the first scan. The major difference between the two sensors was observed at around +0.3 V_Ag/AgCl_, where an increase in the current density was observed with the new sensor. To further investigate this behaviour, the mass change during this transition was examined.

Fig. 3b shows that there is negligible change in surface mass for the used sensor during the potential scan, whilst the new sensor behaves differently – the surface mass decreases in the cathodic domain and remains constant in the passive domain, before starting to increase around the potential where the current density increases in Fig. 3b. The initial decrease, from −1.2 V_Ag/AgCl_ until the corrosion potential is due to desorption of species, such as ions, on the surface of the titanium as described earlier. On the second region that shows the passive domain, the surface mass remains approximately the same, similarly to the used sensor. This was expected since on this region the passive film grows very slowly due to slow transport of metal ions though the oxide film. On the third region that is absent from the used sensor or moved to values above 1 V, both the current and the mass of the new QCM sensor were increased. If this increase is due to breakdown of the film then the mass should have shown a decrease. However, the mass is increased significantly, to approximately 320 ng cm^−2^. A possible explanation is that the oxide layer is changing phase, from anatase which is highly reactive to rutile.^39^ Considering that the mass density of the anatase and rutile phase of titanium dioxide is 3.83 and 4.24 g cm^−3^ respectively,^40^ approximately 3.8 μg of titanium dioxide are required to change from anatase to rutile to explain the observed mass difference (320 ng cm^−2^). This would correspond to a thin film of approximately 7.3 nm of anatase transforming to rutile, which is possible because the typical thickness of oxide film on titanium surfaces is approximately 10 nm.^41,42^ Our speculation is supported by results observed that such effect is irreversible, since it was only observed during the first scan in the titanium-coated QCM crystal, and it affects the electrochemical behaviour of the sensor in the following scans.^43–45^ Despite the unexpected behaviour of the new crystal, potentiodynamic curves confirm that the titanium-coated QCM crystals could represent the bulk titanium substrate in terms of the electrochemical properties, as far as it is used after an initial passivation.

### 3.3 Influence of protein on electrochemical behaviour of titanium

**Fig. 4:**
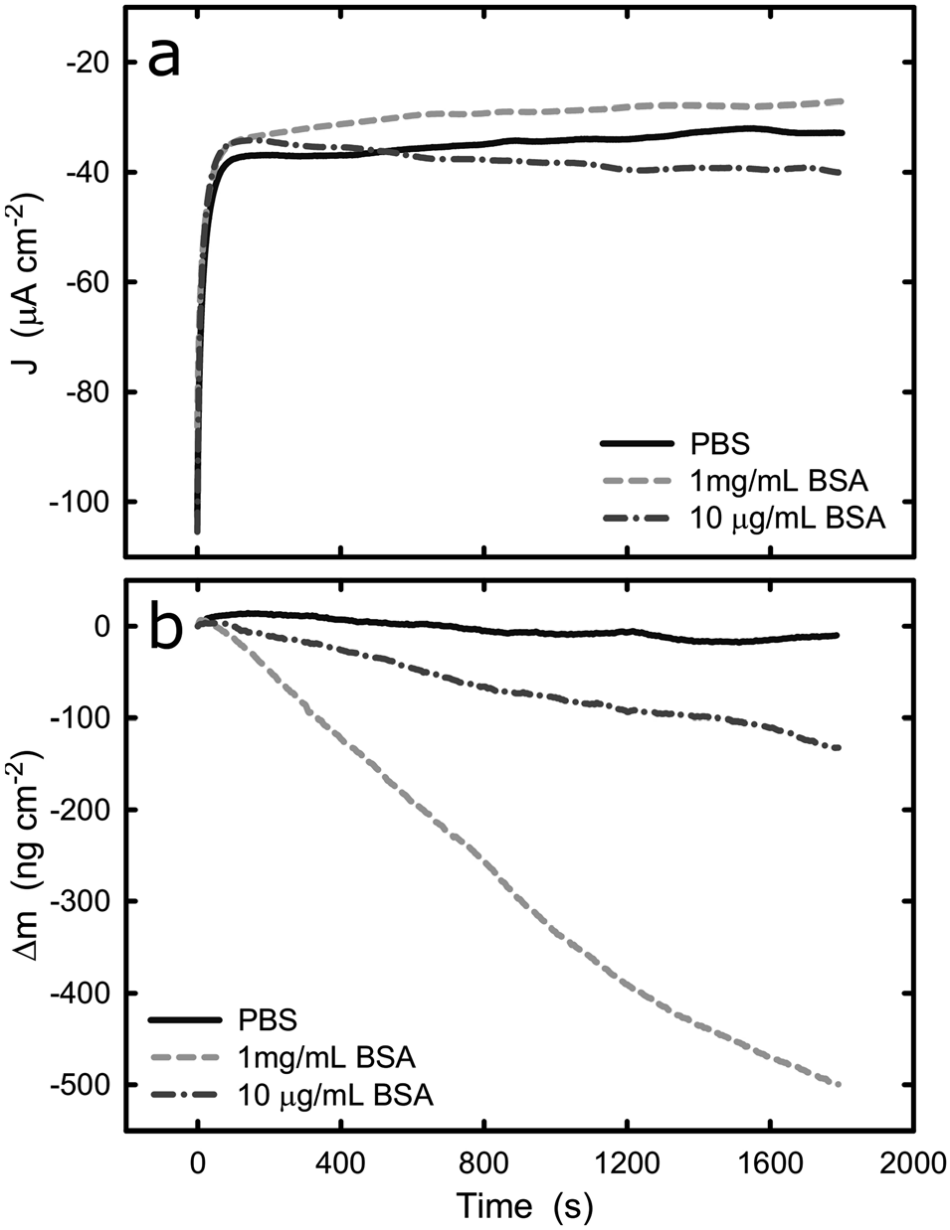
(a) Potentiostatic test of titanium with and without BSA at −1.2 V, (b) evolution of change of mass on the titanium surface with and without BSA at −1.2 V. The graphs represent the average values of *n* = 3 measurements.

Upon the comprehensive characterization of the titanium surface on QCM crystal, the effect of cathodic and anodic potential on the adsorption of BSA onto titanium was examined. The sensors were tested in PBS solution and the change of mass was recorded under cathodic and anodic conditions at two different protein concentrations, 10 μg mL^−1^ and 1 mg mL^−1^. It should be noted that because of the nature of the experimental set-up, an undefined amount of protein molecules is expected to be adsorbed onto the surface of the sensor (in protein solutions) prior to the electrochemical experiment. However, this does not affect the validity of the following measurement because we are considering the change in mass upon application of cathodic or anodic conditions. Fig. 4 shows the current density and mass evolution over time at applied potential equal to −1.2 V_Ag/AgCl_, with or without the presence of BSA (2 different concentrations). For all three solutions examined, the current density decreases rapidly in the first few seconds upon the introduction of the cathodic potential and then reaches a plateau. At the applied potential of −1.2 V_Ag/AgCl_ water and oxygen reduction are the main reactions and in all solutions a current density around −35 μA cm^−2^ was registered. The initial current decrease can be explained by considering that prior to the application of cathodic potential there was an excess of oxygen molecules at the solid/liquid interface. As soon as the potential was applied, the cathodic reactions consumed the oxygen molecules and the rate of the cathodic reactions was limited by the rate which the oxygen molecules are being transported to the titanium surface, resulting in a plateau.

The evolution of mass (Fig. 4b) at the titanium–liquid interface in PBS, converted from frequency monitored by EQCM, shows that the surface mass remains the same during the measurements, indicating that the electrochemical condition has negligible impact on the titanium surface. As a contrast, the surface mass decreases when the titanium was exposed to BSA solutions, with a reduction of approximately 420 ng cm^−2^ and 80 ng cm^−2^ (5 times smaller) for 1 mg mL^−1^ and 10 μg mL^−1^ BSA solutions respectively. Since the effect of titanium in the mass loss is negligible, as confirmed by the experiments in PBS buffer, it is very likely that the mass reduction is due to desorption of protein from the titanium surface. Because the QCM sensor was immersed in protein solution prior to the application of potential, the initial total amount of the adsorbed BSA is unknown. However, previous studies concerning BSA adsorption on titanium show that the surface gets saturated by BSA with density of 710 ± 70 ng cm^−2^.^46^ This suggests that nearly 60% of the adsorbed BSA could be removed over a period of 30 min, as a result of electrostatic repulsion and hydrogen generation during water reduction at −1.2 V. On the other hand, hydration of proteins takes place in a complex manner and have been previously reported.^47^ The hydration depends on the BSA concentration, therefore, it is not surprising that the mass loss in the different solutions leaded to different mass loss. Indeed, if one determines the weight of a water monolayer on the QCM crystal, it corresponds to around 50 ng cm^−2^, which also contributes to the mass loss during the potentiostatic test.^48^

Electrostatic repulsion upon the application of cathodic potential could possibly also play a role in the 1 mg mL^−1^ BSA solution where titanium surface is saturated with BSA. It has been shown that cathode surface is negatively charged if the cathodic reactions are not fast enough to accommodate all of the provided electrons from the ionization of metal atoms.^49^ In contrast, anodic polarization results in electron depletion and thus the surface is charged positively. Therefore, because BSA has an overall negative charge in pH 7, desorption from the cathodic titanium surface could be favoured due to the electrostatic repulsion between surface and proteins.

**Fig. 5:**
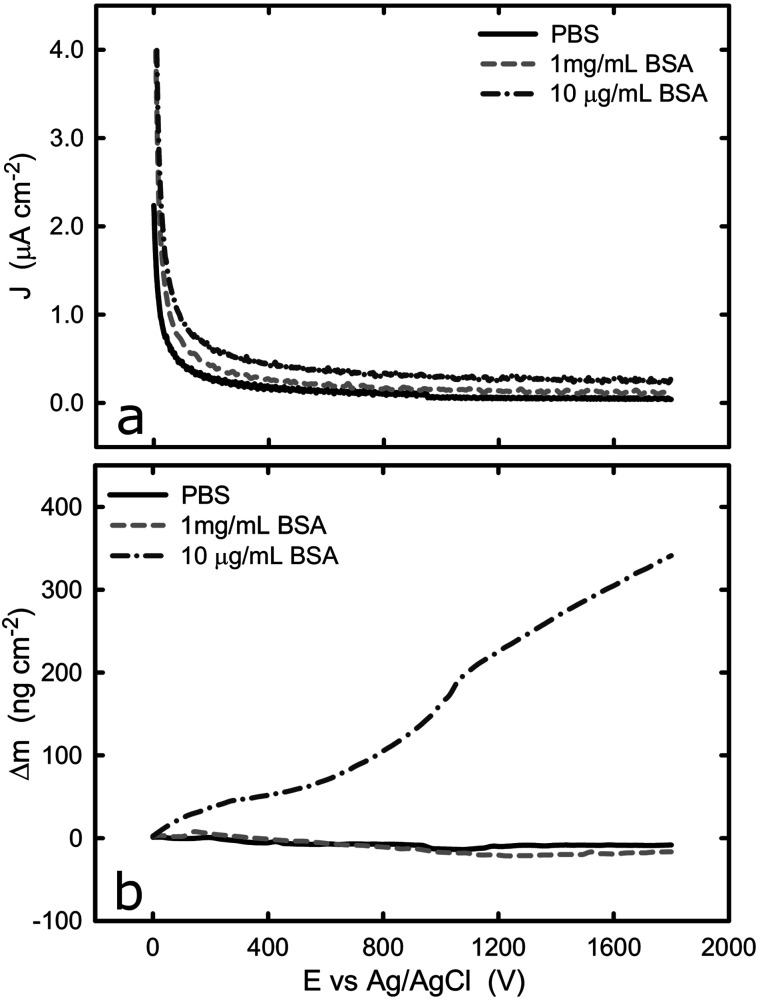
(a) Potentiostatic test of titanium with and without BSA at +1.0 V, (b) evolution of change of mass on the titanium surface with and without BSA at +1.0 V.

**Fig. 6:**
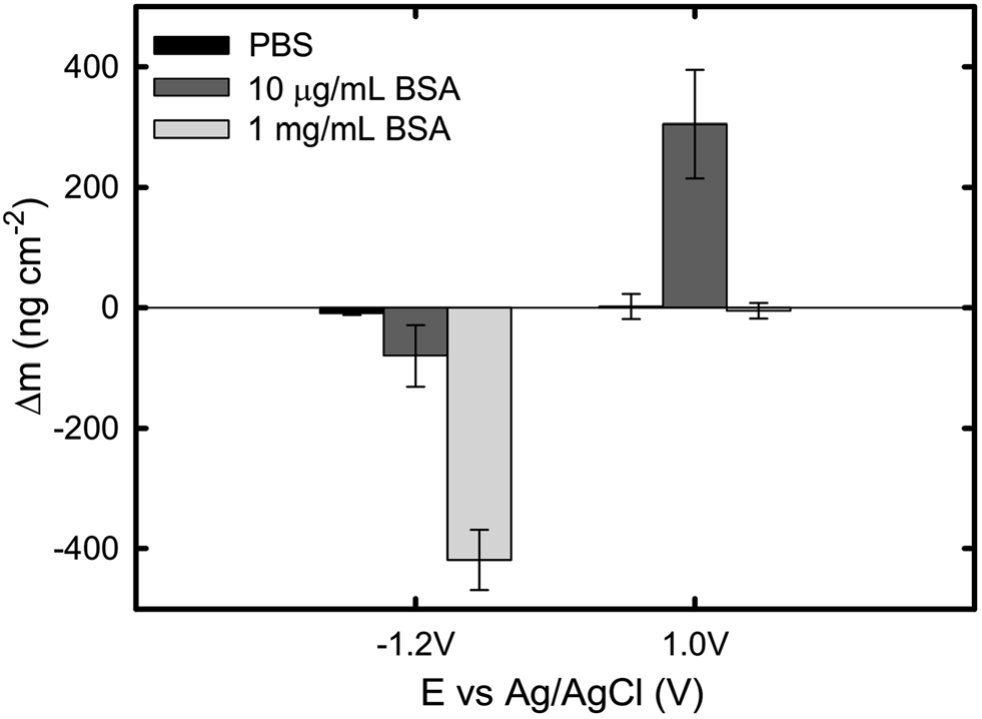
Total change of mass under cathodic and anodic conditions in PBS, 10 μg mL^−1^ BSA, and 1 mg mL^−1^ BSA solutions. Data points represent mean values of *n* = 3 measurements, with the error bar representing the standard error.

The current and mass evolution over time for an applied potential of +1.0 V_Ag/AgCl_ are shown in Fig. 5. Upon application of an anodic potential, the current density exponentially diminishes until it reaches a constant value (Fig. 5a). This is a typical behaviour for current during a passivation process in which the oxide film is formed. The current density at the plateau was found to be around 136 ± 66, 106 ± 52, and 312 ± 103 nA cm^−2^ in PBS, 1 mg mL^−1^ BSA, and 10 μg mL^−1^ BSA solutions respectively. The slight increase in current density in the presence of BSA suggests that the presence of protein could enhance the passive dissolution. The EQCM data shows that the mass remains unchanged in PBS and in 1 mg mL^−1^ BSA solutions, while it increases steadily to approximately 305 ng cm^−2^ (Fig. 6) in 10 μg mL^−1^ BSA. Since the variation in mass remains unchanged in PBS solution, it is likely that the observed increase in mass is a result of BSA adsorption onto the surface of titanium due to the attractive electrostatic interaction between surface and protein at applied passive potential. Also, the fact that protein adsorption was observed only for the 10 μg mL^−1^ BSA solution but not for the 1 mg mL^−1^ BSA solution indicates that the latter was already saturated with protein prior to the application of the anodic potential. The effect of anodic and cathodic conditions in the adsorption of BSA onto titanium surfaces is summarized in Fig. 6. It can be concluded that the application of a cathodic potential results in desorption of BSA with protein dehydration from the titanium surface due to water reduction and electrostatic repulsion, while the application of an anodic potential facilitates adsorption of BSA only if the titanium surface is not already saturated with protein.

## 4 Conclusions

In the present work, both the electrochemical behaviour of pure titanium in PBS buffer and protein solutions, and its influence on the adsorption of BSA onto titanium, were investigated systematically. The electrochemical properties of bulk titanium substrate and titanium-coated QCM sensors show small degree of discrepancy in PBS buffer solution, which are attributed to the difference in their surface roughness. Furthermore, the comparison between new and used QCM sensors suggests that the history of potentiostatic tests has a significant impact on the electrochemical behaviour of Ti surface, and cathodic cleaning is not sufficient to reproduce the same initial electrochemical response.

Applied cathodic potential in the water reduction domain causes desorption of BSA due to dehydration and electrostatic repulsions caused by an excessive amount of electrons on titanium surface and, thus, an overall negative surface charge. In contrast, under anodic applied potential adsorption of BSA was facilitated by attractive electrostatic interactions only when the surface has not already been saturated with proteins. Our work shows that both surface characteristics and the electrochemical environment could have significant impact on protein adsorption to one of the most abundant materials for biomedical applications.

## Conflicts of interest

There are no conflicts to declare.

## Supplementary Material
